# Teacher Creativity: When Professional Coherence Supports Beautiful Risks

**DOI:** 10.3390/jintelligence10030062

**Published:** 2022-09-02

**Authors:** Marie-Hélène Massie, Isabelle Capron Puozzo, Marc Boutet

**Affiliations:** 1 Faculté d’Éducation, Université de Sherbrooke, Sherbrooke, QC J1K 2R1, Canada; 2 Domaine Recherche & Développement, Haute École Pédagogique du Valais, 1890 Saint-Maurice, Switzerland

**Keywords:** creativity, beautiful risks, professional coherence, onion model, professional development, continuing education

## Abstract

Environmental, cultural, and social issues are becoming increasingly complex, and the educational context is no exception to this trend. The relevance of teachers’ creativity in examining situations from different angles, in imagining new approaches, in adapting to the varied needs of students, and in training them so that they too can grasp the teeming complexity seems obvious. However, creativity sometimes seems to be taken for granted among teachers and educational programs leave a gap around this theme. Since the scientific literature tends to show that teachers’ creativity is still little explored in educational contexts, this doctoral research studies its manifestations within a group of teachers enrolled in a professional master’s program in preschool and elementary education (Université de Sherbrooke, Canada). Within the framework of this program, each one elaborates a professional development project over a three-year period. Using a variety of authentic data sources (observations in natural occurring situations, reflective writing by participants, and semi-structured interviews), their creative process is documented and analyzed. This multiple-case study (n = 9) that draws on the concept of creativity as related to that of professional coherence reveals that the pursuit of greater professional coherence not only enables the implementation of creative skills to foster teachers’ professional development, but also elicits beautiful risk-taking.

## 1. Introduction

Several researchers agree that the development and maintenance of creative potential generally contribute to improving the ability to solve problems of various kinds, to adapt or to face complexity more serenely (Capron Puozzo 2016a; Craft 2005; Csikszentmihalyi 1996; Lubart et al. [2003] 2015; Plucker et al. 2004; Robinson [2001] 2017). For teachers who perceive that their profession is becoming ever more complex (Mukamurera et al. 2014), the implementation of their creativity remains an interesting avenue, both for reaping the benefits and providing suitable conditions in the classroom to foster its expression in their students (Beghetto 2005; Cayirdag 2017; Craft 2005).

Officially, *the Quebec Education Program* (Quebec, Canada) mentions that the professional character of teaching is based, among other things, on pedagogical practices that rely on the creativity of teachers (Government of Quebec 2006), without specifying how. The *Référentiel de compétences professionnelles pour la formation du personnel enseignant (Professional Competencies Framework for Teacher Education)* (Government of Quebec 2020) also states that teaching requires “a certain level of creativity” (p. 28). In addition to noting that this formulation is rather nebulous, we observe, by examining the preschool and elementary teacher initial education plans of Quebec universities, that the intentions to teach creativity competencies are scarcely included. In general, the programs do not focus on “the acquisition of skills specific to leadership, ambiguity tolerance, risk-taking or creative problem-solving” (Ouellet 2012, p. 158). As a result, teacher creativity seems somewhat taken for granted since little procedural knowledge of creativity is provided in pre-service development. Inevitably, the barriers to implementing it when it arrives in the school setting persist (Beghetto 2010; Capron Puozzo 2016b).

The scientific literature tends to show that the development of student creativity has been studied from a variety of perspectives, but that there is still an insufficient amount of research on creativity in teacher education (Terzidis 2019). To better understand how creativity translates once initial education is completed and how it can support their professional development, in a master’s program in preschool and elementary education (Université de Sherbrooke)^1^. Over a three-year period, students, in-service teachers, design a professional development project on a topic of their choice, according to their interests and concrete pedagogical or didactic needs. The main intention of the process is to achieve greater professional coherence (Korthagen 2004). This program appears has a favourable context to study teachers’ creativity, since certain factors conducive to its development come together. In particular, the accompanying posture favoured by the trainers’ (a professor and a lecturer) aims to emancipate the students (Boutet et al. 2021; Paul 2012). Believing in reflective practice as a driving force for professional development and changes in practice (Schön 1983) as well as in the value of experience (Dewey 1967; Kolb 1984; Lewin 1951), they encourage risk-taking, openness to new experiences and ambiguity tolerance. Those pedagogical components foster creativity, according to Lubart et al. ([2003] 2015).

Aware that creativity is attractive in educational settings (Cropley 2010) and that it seems good to be defined as a creative person (Karwowski 2009), we were keen to study the phenomenon in a natural context (Guba and Lincoln 1982), i.e., where students are not pressured externally to be creative, not being evaluated in this regard. Broadly, we are interested in the contribution of creativity to professional development, but for this article we focus on one of our research sub-questions which concern the risk-taking that students took in developing their project. What is the nature of the risks (Beghetto 2019) that were taken? What place do they take in view of their professional coherence (Korthagen 2004)? What level of creativity (Beghetto 2019) did the students express in the development of their project? This article brings forward some of the answers.

## 2. Conceptual Framework

To introduce the conceptual framework, it is important to first clarify the relevance of professional coherence, specified by Korthagen’s (2004) onion model, as it is foundational to the program’s approach. The concept of creativity and one of its influential conative factors (Lubart et al. [2003] 2015), taking beautiful risks (Beghetto 2019) is then brought into light to provide a complementary scientific insight for this professional development space.

### 2.1. Professional Coherence

For the teaching community, which daily modulates its interventions and practices according to singular situations by assuming the great complexity of the school environment (Wanlin and Crahay 2012), reflective practice seems to be a precious, even essential asset (Boutet et al. 2016; Guillemette and Gauthier 2008; Lison 2013; Perrenoud 2001, 2004; Tardif et al. 2012; Vacher 2015; Wentzel 2010). Since the work of Schön (1983), it has become increasingly important in research and devices on professionalization. He points out that competent practitioners generally know much more about their practice than they can say about it, hence the interest in getting them to seek out all the knowledge buried in the depths of their professional action.

**Figure 1:**
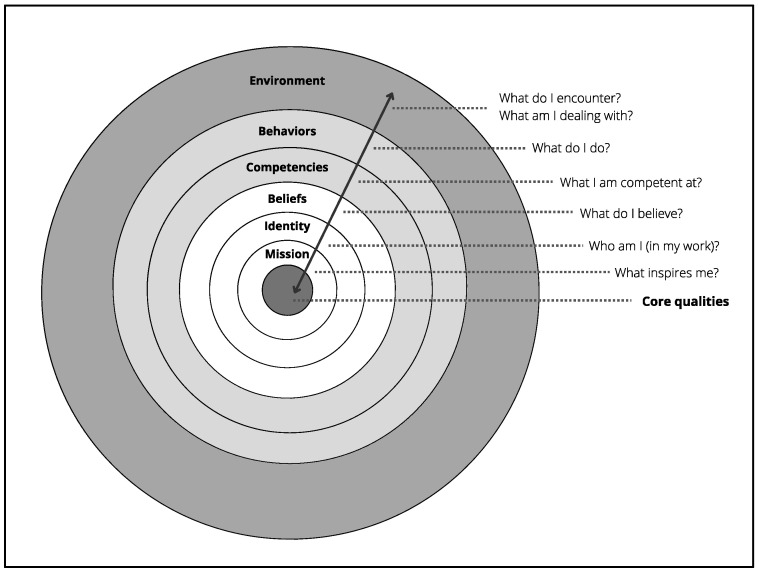
Onion Model—Level of Change (Korthagen 2004).

Engaging in the program, students are prepared for the development of their project, by being led to explicit the elements that compose their professional universe based on Korthagen’s onion model (Korthagen 2004) (Figure 1). Resting on positive psychology, this model of reflection allows to examine each of the layers that are intimately related to go beyond a purely cognitive reflection (Korthagen 2017).

Thus, with the help of a framework, each person puts into words specific personal and professional components that influence each other: their fundamental qualities, their deep mission (why they chose this profession, what inspires them), their identity (who they are as a teacher), their beliefs (what matters to them and underlies their choices), their skills (what they feel capable of), their behaviours (the concrete actions they take) and the environment in which they navigate (and which influences them daily). The students can then become aware of what is likely to hinder the coherence between their internal and external factors. In this regard, Dewey (1947) reminds us that during moments of rupture, questions come up and consciousness, “whose role consists precisely in readjusting experience, a critical and creative role, an essential role” (p. 6), emerges. The feeling of uneasiness felt in the rupture then creates an imbalance. Returning to one’s core mission during difficult times would support and propel the teacher’s commitment to change (Day 2004) as it gives meaning to the profession. This step back also allows practitioners to realize that they hold the power to prevent certain constraining factors in determining their behaviours; this awareness of choice contributes positively to autonomy (Korthagen and Vasalos 2005).

### 2.2. Creativity

The need to adapt to the environment is the basis of the creative act, since without an imbalance with the world around, individuals will not feel the need to exercise their creativity (Csikszentmihalyi 1996; Vygotsky 2004). The work of Terzidis and Darbellay (2017), establishes the relevance of supporting creativity at the service of professional development, as an avenue to ensure its sustainability. Returning to core quality, identity, and professional beliefs (Korthagen 2004) beforehand serves as an anchor for the creative process. Several definitions of creativity exist, and our research is based on one proposed by Lubart et al. ([2003] 2015), which is “the ability to produce work that is both novel and appropriate to the context in which it occurs” (p. 23). Originating from differential psychology, it is agreed upon by several researchers such as Amabile (1983), Beghetto (2021), Capron Puozzo (2016a), Robinson ([2001] 2017), Runco and Jaeger (2012), or Sternberg and Kaufman (2010) who also recognize the novelty and relevance necessary as criteria for creativity. Supported by several works, including those of Corazza (2016), Botella and Lubart (2019) bring forward the dynamism perspective for the creative process. This approach incorporates, among other things, interactions with the environment and the perpetual motion of the process, even after production has been completed.

To avoid reinforcing the myth that creativity is only about a handful of privileged individuals revolutionizing history (Plucker et al. 2004; Robinson [2001] 2017), Kaufman and Beghetto (2009) propose the Four C model distinguishing four levels of creativity:

- *mini-c* or self-recognized creativity (exploration activities through new experiences)
- *little-c* or creativity recognized by people in the immediate environment (more thoughtful, leading to productions that are out of the ordinary)
- *pro-c* or creativity recognized by experts in the field (people who have become experts in their field)
- *big-C* or legendary creativity (enduring creativity recognized on a very large scale)

This model provides a framework for including creativity in school curricula and helping students develop their creativity at higher levels (Beghetto and Kaufman 2013). To make it even more appropriate in the school setting, Beghetto (2019) refines it by removing the *big-C* and adds *surprising little-c* (when the student meets the criteria in a surprising, original, or significantly different way) and *no-c* (when there is no creative attempt). By incorporating them into the continuum, they can help students better situate and challenge themselves. It is this modified model that we adopted for our study. 

To understand the leverages and obstacles that can influence or inhibit the creative process, the multivariate approach (Lubart et al. [2003] 2015) provides a systemic view that binds four types of influencing factors: cognitive, conative, emotional, and environmental factors. Cognitive factors refer to the knowledge and intellectual abilities that underlie creative thinking. Conative factors, which refer to the ability to induce intellectual energy in the performance of a task to achieve a solution or completion (Reitan and Wolfson 2000), specifically relate to perseverance, ambiguity tolerance, risk taking, openness to new experiences, and individualism (Besançon and Lubart 2015). Emotional factors are based on the affective state inducing positive sensations such as well-being, appeasement, joy or negative such as stress, frustration, or anger (Capron Puozzo and Botella 2018). The environmental factors refer to the contextual elements in which the creative process takes place.

Through all these variables, creativity is, therefore, complex, and Beghetto (2005) denotes that the most valuable form of creative expression often occurs within the boundaries of real-life structures, rules, and norms. In the school setting, teachers who take more risks and initiatives in difficult situations are more likely to demonstrate creative teaching behaviours, as they accept constraints rather than challenge or attempt to eliminate them (Cayirdag 2017). They then manage to turn adversity into an advantage (Kaufman and Gregoire 2016).

### 2.3. Taking Beautiful Risks

Risk-taking has been identified as influential to the creative process, as creative ideas differ from the usual ideas of a community (Csikszentmihalyi 2006; Lubart et al. [2003] 2015; Sternberg and Lubart 1995). Tyagi et al. (2017) further demonstrates that it is social risk-taking that is linked to creativity. People who are considered creative are more likely to present their ideas and creative outputs to a group for evaluation. Therefore, they show a high level of risk, as it is possible that some or all members of the group may reject the idea or production in question. Research has shown that school environment is still often seen as rigid and not very open to risk-taking (Besançon and Lubart 2015; Csikszentmihalyi 2006). Ouellet (2008) adds that the dominant conventional views of society, the education system and professional environments impose significant challenges to the development of a creative societal culture. For fear of displeasing colleagues, managers or parents, many teachers prefer to keep their ideas, however attractive, to conform, to blend in, rather than risk being disturbed or rejected (Huret 2014). The pressure of the environment leads many to look for quick, short-term solutions to patch things up rather than to address the real causes of the problem and solve it (Korthagen and Vasalos 2005). However, creative risk-taking could contribute finding innovative ways to solve problems, for the benefit of students or the school.

At the root of all historical advances, creativity can have very positive repercussions, but also negative ones, if ill-intentioned. Amabile (2018) reminds us that “it is only by combining creative capacities, strong passions, and conducive environments with equally strong moral values that we will be able to harness the power of creativity for the good of humanity and not its destruction” (p. 13). Thus, the values underlying creativity matter. Beghetto and Anderson (2022) abound in this sense by suggesting an approach to creativity that aims to a beneficial contribution to others. Beghetto (2019) calls for taking “beautiful risks” (p. 2) which he distinguishes from good and bad risks. The good risk occurs when the potential benefits outweigh the potential costs and the bad risk, conversely, presents potential costs higher than the possible benefits. Beautiful risk occurs when the potential to make a positive contribution to others outweighs the potential costs. Beghetto gives examples of a teacher who shares a pedagogical practice she or he has developed with colleagues, who accepts the uncertainty caused by a new and complex challenge, or who continues to believe in a student’s potential even though other colleagues have stopped doing so. To plan such a contribution, one must first ensure that the planned action can benefit others, that possible hazards have been addressed, and finally, that the plan is carried out. 

In the context that we studied, the participants are asked to design a professional development project that builds on core qualities “considered as the driving force of productive teacher learning, and also as fundamental to the development of competencies” (Korthagen 2017, p. 396) as well as a positive mission and beliefs of teaching (Korthagen 2004, 2017). This process is part of a creative one, according to Lubart et al.’s ([2003] 2015) definition of creativity, as students are challenged to update their practice (novelty) while respecting the elements of their context such as the program, their pupils, and the constraints of their environment (relevance). It is therefore a question of seeing whether the students took creative risks to harmonize their coherence.

More specifically, our research sub-objectives regarding this conative factor aim to determine the nature of the risks taken in the development of the project (based on Beghetto’s three criteria), to identify the components of the onion model (Korthagen 2004) that are consistent with taking beautiful risks and to specify the level of creativity expressed by the students, according to Beghetto’s scale.

## 3. Method

To describe and understand in depth the issues of creativity, particularly concerning the taking of beautiful risks in a real situation of professional development, that is, in a research space where the phenomenon and the context merge (Arbarello 2011; Merriam 1988; Miles and Huberman [1994] 2003; Yin [1984] 2018), the multiple-case study was privileged. This methodological choice was appropriate for our exploratory research, in accordance with Stake (2006) and Yin ([1984] 2018) who state that case studies can explain, describe, or explore events or phenomena in their singular context. The choice to cumulate several cases was intended to take advantage of the possibility of comparison and add to the scientific rigour (Creswell [2006] 2013; Gagnon [2005] 2011; Yin [1984] 2018). Without testing or corroborating a hypothesis, the analytical approach of this multiple-case study is deductive, as it draws on existing theoretical frameworks (factors of creativity and creative risk-taking), allowing for the delineation of cases (Alexandre 2013).

The sample (n = 9) is drawn from a cohort of students, in-service teachers, enrolled in a three-year master’s program in preschool and elementary education (Université de Sherbrooke, Canada). Initially, 17 people in the group expressed interest in participating in the research project at the time of the first contact in April 2021, that is, once the master’s degree was completed. Fourteen individuals completed and returned the ethical consent form, but only nine followed up to participate in a semi-structured interview (Savoie-Zajc [1984] 2016). This purposeful sampling (Creswell [2006] 2013) allows nonetheless for an optimal number of cases to conduct a multiple-case study, conforming to Stake (2006) and Yin ([1984] 2018) who suggest between 4 and 10 as an appropriate sample. This sampling strategy allows for consistency in case studies, as the research object is specific to this continuing education context (Hamel 1997; Merriam 1988; Pires 1997; Savoie-Zajc 2007). Moreover, the sample’s representativeness in terms of the gender distribution in teaching can be observed: the sample includes eight women and one man, which corresponds well to the current portrait of the school environment, where in 2017–2018 men represented approximately 12% of the teaching staff (Government of Quebec 2019).

**Figure 2:**
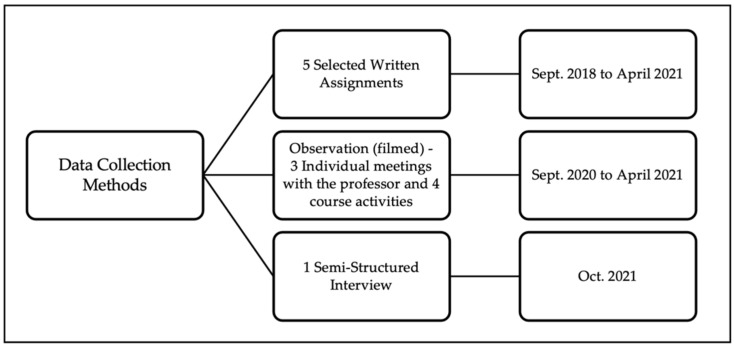
Data Collection Methods.

In order to achieve the richest and most rigorous multiple-case study possible, three qualitative data collection methods were used (Creswell [2006] 2013; Merriam 1988; Stake 2006; Yin [1984] 2018) (Figure 2): (1) written assignments from all three years of the educational program were selected; (2) individual meetings with the professor and course activities that were filmed during the third year; (3) semi-structured interviews were conducted six months after the end of the master’s program. 

Data collection methods covering a three-year period allows for some “temporal thickness” (Leplat 2002, n.p.), a strength of the research design. Through various studies, Cropley (2010) identifies some inconsistencies in the teaching community’s discourse about creativity. Runco and Johnson (2002) also point out that it looks good for teachers to say they value creativity in their classrooms even if, in fact, they do not necessarily do so. Given that this type of gap often found between espoused theory (what individual claims to follow) and theory-in-use (deducted from the action) (Argyris and Schön 1999), observations to capture live data seemed necessary to corroborate the reported practices and intentions mentioned in the written work. COVID-19 pandemic made it even easier to access the natural context without interfering with it, since to provide quality support, the trainers recorded individual meetings and class sessions. The semi-structured interviews allowed for a privileged return on the experience (Savoie-Zajc [1984] 2016) and mainly identified the students’ perceptions of creativity through the development of their project. To personalize each of the interviews, a chronological pre-analysis of the training course of each participant was conducted (Bardin [1977] 2013), with the goal of designing a specific timeline. This tool supported for the targeting of moments, recalling details already far away, and again decreasing the desirability bias, by relating the reflection to observable facts that happened during the program. In addition to ensuring the triangulation of data, using this variety of data collection methods helped to reduce the inherent biases and limitations of each (Mucchielli 2002) and increase the level of objectivity.

To process the collected data, a qualitative content analysis according to Bardin ([1977] 2013) was completed in three steps: (1) pre-analysis, (2) exploitation of the material and (3) processing of the results including inference and interpretation. In addition to preparing for the semi-structured interviews, the pre-analysis granted for the constitution of the corpus, following the rules of completeness and non-selectivity (considering all documents that justify its rigour), representativeness (analyzing a sample), homogeneity (comparability of documents), and relevance (ensuring that the documents are adequate as a source of information). 

Subsequently, a categorical analysis, consisting of cutting the text into units of meaning and classifying them into categories with a common character (Alexandre 2013; Bardin [1977] 2013), was carried out based on Lubart et al.’s ([2003] 2015) creativity factors (cognitive, conative, emotional, and environmental). Chronological analyses, based on in-class situations, written works, and interviews verbatim, allowed us to respond to our research objectives. To ensure a thorough analysis of each data source, Gaudet and Robert’s (2018) vertical analysis proposal was chosen. To do so, the first step is to accomplish a contextualizing condensation by identifying the material and documenting its production context. In a second step, a semantic condensation is performed with the aim of reducing the material in terms of meanings derived from the research questions. Several readings of the material or viewings of the recordings were necessary to identify relevant themes and make links to the scientific literature on creativity before reducing, to avoid losing important content. Moreover, following Miles and Huberman ([1994] 2003), a within-case analysis was carried out to provide a full description for each of the nine cases. Finally, the cases were linked through cross-analysis to extend the generalization and deepen the understanding and explanation of the phenomenon of creative risk during the design and implementation of the professional development project.

## 4. Results

In line with the sub-objectives of this doctoral research, this section summarizes what the participants mentioned, orally or in writing, about the nature of the risks taken in the development of the project (according to Beghetto’s three criteria), the components of the onion model that are compatible with taking beautiful risks and the level of creativity they expressed.

### 4.1. Taking Beautiful Risks for the Professional Development Project

The first finding that emerges from the analysis concerns the taking of beautiful risks, the main element of interest in this article. To be identified as such, this type of risk must meet three criteria (Beghetto 2019): Is there a potential benefit to others? Have potential hazards been addressed? Were actions taken? (Table 1) Regarding the first question, participants specified the goals of their project in a written assignment during the 3rd year (December 2021), which made it clear how their production could benefit others (their students, their school, the school community in general). The analysis of this artifact was supported by what was said during the semi-structured interview. Concerning the potential hazards, qualitative data collected during a team activity conducted in October 2021, reported on their occurrence. They were also corroborated by excerpts of verbatim from the semi-structured interview. Finally, the meetings with the professor at the end of the course and the public presentation of the project in an online symposium were clear evidence of whether the project was put into action or not.

In conformity with Beghetto’s criteria, the Table 1 shows that 7 of the 9 cases took a beautiful risk to design and implement his project. For example, as early as Year 1, Case 9 expressed wanting to support students’ motivation in his music class. 

Extract of verbatim

*For me, motivation is the focus point. I was a student who didn’t like school, I found it boring as hell, but I loved learning, so it was clear from the start that my project revolved around motivation.*

After having thought of several ideas (putting on a show, collaborating with a high school teacher, making a recording of traditional songs, having a musical tournament between two groups from different schools…) and having dealt with several constraints (coping with the departure of a sick colleague, dealing with the pandemic, having very limited audiovisual equipment…), an entrepreneurial project was launched so that the students could make the choices for themselves. They chose to produce a video clip that denounced sexism. Both devices (entrepreneurial approach and producing a video clip) were novelty to him, and he was curious to explore them. With the support of an expert in the entrepreneurial approach and a colleague (the lead teacher of the target group), he was able to foresee certain pitfalls (choosing the group of students, finding the missing material, organizing time outside of class…) and complete the project.

Two of the nine cases (Cases 2–6) did not meet the three criteria for a beautiful risk. They were certainly having a project that benefited their pupils and for which they had addressed the potential hazards. However, being absent on maternity leave towards the end of the course, a leave that was still in effect at the time of their participation in the semi-structured interview, they were unable to put their planning into action at the time of data collection. A clarification is necessary for Case 6. At the beginning of the master’s training, the participant was teaching in an international program where the academic curriculum includes heavily built teaching modules. She had expressed in the 2nd year that she felt uncomfortable in this work environment, despite a pleasant and stimulating climate. Following the development of her project, which consisted of building an outdoor education plan, she made the choice to change school in preparation for her return to work. This new environment would permit her to teach where fewer academic constraints existed and, moreover, was geographically located just a stone’s throw from a wooded area. So, in this case, it can be considered that one step for action was taken. 

### 4.2. Components of the Onion Model Related to the Professional Development Project

Still concerning Case 6, it is important to note that this change of school is congruent with some of her professional beliefs, i.e., the importance of cultivating the well-being of her students and taking time to avoid creating stress (Table 2—The excerpts regarding the onion model are from the very first assignment in the program (December 2018) when participants were asked to paint a picture of their professional reality). This precision leads us to a second result: in general, the beautiful risks were taken in coherence with some components of their onion model. Case 5, for example, expressed her mission as offering its students stimulating and varied activities to support the pleasure of learning, while being concerned with encouraging the development of their critical thinking. She chose to plan a website around three ethical topics (overconsumption, environment, and differences) in a multidisciplinary approach. By creating stimulating interrelated activities that integrated art projects, children’s literature, math problems, or science experiments, she was able to explore these themes in a more articulate and in-depth way. For Case 4, the importance of collaboration and sharing among colleagues was a common thread in her onion model and the primary intent of her project was to support in service teachers by providing a well-organized resource bank.

Emotional factors seem to have played a role in beautiful risks taking, especially for the students who carried out their project in real time, within their school (Cases 3-7-9). In keeping with their professional beliefs, they were confronted with some colleagues who did not have the same. For Case 3, who wanted to use different evaluation methods than her colleagues, the pressure from the environment was heavy. In a meeting with the professor (December 2021), she cried and mentioned that her sense of personal effectiveness was no longer as strong. The professor suggested that this painful emotion be seen as rich material for reflection and use it to further strengthen the foundations of her project. During the semi-structured interview, she said that this emotional moment was a turning point to find a solution and take action. She illustrated this with a metaphor of rafting:

Extract of verbatim

*In the beginning, you must paddle and it’s very hard, but when you catch the rapids, it’s so fun! And even today, I would say that I’m still in the fun part, because it’s not over, it’s still going on!*

This beautiful risk taken for the benefit of her pupils has helped to anchor her beliefs and she said she felt much stronger, grounded, and she is now able to defend her choices in front of her colleagues. This excerpt also unveils the dynamic side of creativity, as her project is still in movement, corroborated by other statements. This dynamic trend was found in five other cases (1-5-6-7-9), who mentioned that their project was still evolving.

### 4.3. Level of Creativity of the Professional Development Project

The third finding concerns the level of creativity. Recall that to be creative, the idea or production must be both new and appropriate (Lubart et al. [2003] 2015). Therefore, from a perspective of coordinating some components of their onion and the objectives of their project, students demonstrated creativity by introducing something new and appropriate (respecting the Quebec School Training Program (Government of Quebec 2006) and the constraints of their school environment) to their practice or school. In light of some exchanges with the professor and the semi-structured interview, we noted the presence of mini-c (self-recognized creativity) for all cases except one (Case 8) for whom the element of novelty was not present.

Extract of verbatim

*Was the project creative for me? No, because there weren’t many new things that enhanced my practice; the project allowed me to better organize what I was doing and be able to share it with others.*

Nonetheless, she says that the knowledge she has gained about neuroscience has confirmed the pedagogical choices she was instinctively making. In addition, the creativity of her planning was recognized by colleagues in her school and members of the teaching community who attended the conference (little-c). In this regard, according to the nine individuals, they all perceived their project to be creative for their school. Analysis of the data revealed that by living their project in their community (Cases 3-7-9) or by sharing it with their school colleagues and at the conference (Cases 1-2-4-5-6-8), the feedback received by peers seemed to confirm their perception. For example, the website produced by Case 4 was shared by the educational advisor in professional insertion to new teachers in her service center. The outdoor planning of Case 6 was praised by conference participants, and she subsequently incorporated into an outdoor education committee at her school service center. While designing their project, they conducted a variety of research to support their beliefs and pedagogical choices, to form their ideas, or to see what already existed. Knowing they would have to present their project at a conference and would be invited to disseminate their production, a general concern not to create something that already existed was present in all cases. As a result, they produced something that was creative for their school environment.

Finally, students noted that interactions with peers and accompaniment from the trainers were beneficial in increasing their level of creativity (environmental factors). In six cases, teams’ activities during the program to discuss their project was one of the main leverages of their process. Input from school colleagues or even positive comments from parents helped four cases to push their creativity further. Case 7 felt that having a committee of colleagues was positive in overcoming her limitations, despite some constraints related to her school context that caused her project to deviate.

Extract of verbatim

*It was a “collective” creativity […] if I had been on my own, I would have just had my own ideas, but the fact that I was working with other people, the ideas germinated and went even further.*

For eight of the nine cases, the program approach also became one of the most significant leverages for their creativity.

Extract of verbatim (Case 8)

*We always felt the safety net. “Go ahead and explore, you’re the leader of your project, but if you have a need, were always here.” I could really feel the confidence in us.*

Analysis of the data revealed a recurrence of this sense of security across different artifacts, particularly in the team meetings and the semi-structured interview.

## 5. Discussion 

The results of this exploratory doctoral research report that most of the students dared to take beautiful risks to design and implement a professional project, which, it should be remembered, was aimed at harmonizing their professional coherence. Given their personal context (absent on maternity leave), two cases were unable to take action. This was not by fear, but a circumstantial cause. For Case 6, who changed schools before returning to work, perhaps it is a good risk in preparation for a beautiful risk? It would be interesting to see if, they will put their project into action when they return to the classroom.

Three of the cases, the more experienced ones (Cases 3-7-9) took greater risks by developing their project in real time and involving colleagues in their school (Tyagi et al. 2017), rather than opting to share planning or resources after the project was completed. Following the potential benefits of continuing professional development (Gaudreau et al. 2012; Mukamurera et al. 2019; Paquay et al. 2012; Uwamariya and Mukamurera 2005), it is possible that this willingness to take such risks was facilitated by their greater professional stability, effectiveness, and teaching knowledge. In addition, data analysis shows that these individuals have a positive view of their creative potential and were disposed to take this risk (Beghetto et al. 2021). 

In terms of levels of creativity expressed, students went beyond the mini-c to express a little-c. The results of the analyses do not allow us to determine whether there were any surprising little-c, as it would have been necessary to interview the course instructors to know whether some students met the project process criteria in a surprising way (Beghetto 2019). The same applies to the pro-c, as a much broader investigation would be needed to find out whether some projects were recognized by experts in the educational field. Also, in wanting to improve their professional coherence, the beautiful risks involved seem compatible with the dynamic process of creativity (Botella and Lubart 2019, the project being always in motion for most of them. Therefore, it is possible that it will eventually evolve to a higher level of creativity. It should be remembered that teachers play an important role in establishing the necessary conditions in the classroom to allow for creative learning, while also serving as role models (Aschenbrener et al. 2010; Besançon and Lubart 2015; Capron Puozzo 2016a; Jeffrey and Craft 2004; Zhou 2003). Furthermore, the *Référentiel de compétences professionnelles pour la formation du personnel enseignant (Professional Competencies Framework for Teacher Education)* (Government of Quebec 2020) state that since the turn of the century, social expectations of the school and the teaching profession have multiplied, while the ecological, social, cultural, and economic environment in which new generations are born, grow up and develop has become more complex. Hence, cultivating creativity seems to be a valuable path.

Certain conditions, having contributed to the taking of beautiful risks and the overcoming of a mini-c, lead us to the words of Lucas (2001), who proposes four keys to foster creative learning:

- The need to be challenged by setting goals while being accompanied to overcome them—students designed a project to update their professional practice responding to specific objectives, while being supported by trainers and peers
- The elimination of negative stress—trainers fostered safe space (Rogers 1984), adopted an emancipatory accompaniment posture (Boutet et al. 2021), welcomed the most of emotions in the service of creativity (Audrin et al. 2020)
- The ability to live with uncertainty—this program master is spread out over three years and there are periods of incubation and floating
- The importance of receiving feedback (individual meetings with the professor, team activities, feedback on written work).

Even though this professional master’s program in preschool and elementary education is not focused on creativity, students naturally expressed their creativity to harmonize certain components of their professional coherence given the educational context that provided favourable conditions. It should be noted that Lubart et al.’s multivariate approach to creativity (Lubart et al. [2003] 2015) is intended to be systemic, entangled, we can see the contribution that emotional factors (e.g., welcoming emotions and using them for creativity), cognitive factors (e.g., setting goals, deepening one’s knowledge), environmental factors (e.g., accompaniment, safe space, feedback), or other conative factors (e.g., uncertainty tolerance) can make beautiful risk taking. Since taking beautiful risks is about daring/caring for others, is this a purely conative factor or does the necessary empathy make it also an emotional factor?

It is worth recalling that reflective practice is at the heart of this master’s program. This quote from Schön (1983) may lead to a parallel between creative and reflective postures:

the practitioner gives an artistic performance. He responds to the complexity, which confuses the student, in what seems like a simple, spontaneous way. His artistry is evident in his selective management of large amounts of information, his ability to spin out long lines of invention and inference, and his capacity to hold several ways of looking at things at once without disrupting the flow of inquiry.

(p. 129)

Is there not an interesting parallel here with creativity process? The relevance to further this research project by pursuing the work of Capron Puozzo and Wentzel (2016) on reflexivity and creativity would certainly be of interest. Moreover, by linking them with the aim of improving teachers’ professional coherence, perhaps they would find innovative ways to feel more well-being at work (Kenny et al. 2021).

One of the fields that constitutes Lubart (2017) 7 C’s model is the importance of the collaboration in the creativity process. The main leverages for project development through the accompaniment and exchanges with the other students were significantly noted. The other C’s of the model (Creator, Creating, Contexts, Creations, Consumption, and Curricula) can allow researchers who study some of these invariants to link them together for further research. This is another interesting way to consider for our future research.

Based on core qualities, the internal spheres of their onion model referred to beliefs and educational practices at the service of the child and their development (interest in neuroscience, concern for considering children in their entirety, adopting caring practices, forging an attachment bond with each child, considering their motivation, helping them to develop emotional, social, and interdisciplinary skills…). All these positive elements and the risks associated with the implementation of the project seem to be pointing towards the proposal for positive creativity (Beghetto and Anderson 2022; Sternberg and Chowkase 2021). In addition to proposing creative devices and providing a framework conducive to creative learning, it would be a matter of teaching creativity as a skill, making explicit what beautiful risk-taking (Beghetto 2019) and positive creativity (Beghetto and Anderson 2022) are, to go even deeper and further. 

As this is a purposeful sample, it is possible that these volunteers may have personality traits that are favourable to the research topic (e.g., openness to experience and risk-taking) which may have led to some bias. Since the study was conducted in a very specific training context, reproducibility remains low. Since the study was conducted in a very specific training context, reproducibility remains low. This limitation could be overcome by studying a whole group, or by opting for purposeful maximal sampling (Creswell [2006] 2013) to select more typical cases that might allow larger variations to emerge. Nevertheless, this study sheds new scientific light, as we did not find any research that combined beautiful risk-taking and professional coherence. If taking beautiful risks is compatible with certain components of the teacher’s professional world, then educational benefits may be possible for both the students/school environment (recipients of the beautiful risks) and the teacher. Other initial and continuing education contexts could also be considered for supplementary and broader research on professional coherence supported by a creative process.

## Figures and Tables

**Table 1:** Professional Development Project: Beautiful Risks?

Cases—Professional Development Projects Outlines	Beautiful Risks (Beghetto 2019)		
	Potential Benefits to Others?	Addressed Potential Hazards?	Take Action?
Case 1 Executing and sharing an annual personalized planning in social sciences	✓	✓	✓
Case 2 Annual planning in social sciences without workbooks	✓	✓	X
Case 3 Evaluation in a meaningful way for all (conference)	✓	✓	✓
Case 4 Tools to support teachers in professional insertion (website)	✓	✓	✓
Case 5 Teaching ethical topics using multidisciplinary approaches (website)	✓	✓	✓
Case 6 Outdoor education planning (website)	✓	✓	X
Case 7 Implementation of an annual theme school based on neuroscience	✓	✓	✓
Case 8 Planning in mathematics using neuroeducation approach (website)	✓	✓	✓
Case 9 Music for school motivation (entrepreneurial project)	✓	✓	✓

**Table 2:** Professional Development Project and Onion Model Components Related.

Cases—Professional Development Projects Outlines	Some Components of the Onion Model Related to the Project (Korthagen 2004)
Case 1 Executing and sharing an annual personalized planning in social sciences	Want to personalize practice; Very organized; Importance to share among colleagues
Case 2 Annual planning in social sciences without workbooks	Passion for history; Ability to plan; “Using workbooks is not an optimal practice”
Case 3 Evaluation in a meaningful way for all (lecture)	Want to see children happy to learn; Importance of differentiation; reduce stress related to assessments
Case 4 Tools to support teachers in professional insertion (website)	Collaboration;Share with colleagues; Build on each other’s strengths
Case 5 Teaching ethical topics using multidisciplinary approaches (website)	Offering stimulating and varied activities; Encourage critical thinking
Case 6 Outdoor education planning (website)	Taking time; Promote student well-being; Teaching in a positive climate
Case 7 Implementation of an annual theme school based on neuroscience	Promote overall health; Foster a safe environment; Has good leadership
Case 8 Planning in mathematics using neuroeducation approach (website)	Believe in the potential of each student; Very good knowledge of the program
Case 9 Music for school motivation (entrepreneurial project)	Contribute to students’ well-being; Help them to develop passions; Good adaptability

## Data Availability

The data presented in this study are available on request from the first corresponding author and are not publicly available to ensure the privacy of the participants and to comply with the instructions of the ethic committee that approved this study.

## Note

1 In Quebec (Canada), a Master of Education program is a continuing education program at the graduate level. People who register for it do so on a voluntary basis.
